# The Impact of Exogenic Testosterone and Nortestosterone-Decanoate Toxicological Evaluation Using a Rat Model

**DOI:** 10.1371/journal.pone.0109219

**Published:** 2014-10-10

**Authors:** Romeo Teodor Cristina, Flavia Hanganu, Eugenia Dumitrescu, Florin Muselin, Monica Butnariu, Adriana Constantin, Viorica Chiurciu

**Affiliations:** 1 Pharmacology and Pharmacy Departments, Banat's University of Agricultural Sciences and Veterinary Medicine “King Michael of Romania” from Timişoara, Timiş, Romania; 2 Toxicology Department, Banat's University of Agricultural Sciences and Veterinary Medicine “King Michael of Romania” from Timişoara, Timiş, Romania; 3 Biochemistry Department, Banat's University of Agricultural Sciences and Veterinary Medicine “King Michael of Romania” from Timişoara, Timiş, Romania; 4 National Reference Laboratory for A3 Steroids, Constanţa, Constanţa, Romania; 5 Drugs Production Department, Romvac Company, Voluntari, Ilfov, Romania; Baylor College of Medicine, United States of America

## Abstract

The impact of exogenic testosterone (T): 1.5 and 3.0 mg/kg.bw) and 19-nortestosterone 17-decanoate (ND): 1.5 and 7.5 mg/kg.bw) in castrated male rats was evaluated based on: (a) weight increase of the androgen target tissues, respecting the Hershberger methodology; (b) the 17α and β-testosterone, 17 α and β-estradiol and 17 α and β-nortestosterone levels using the GC-MS/MS technique; and (c) observation of the serum free thyroxine levels (T_4_). Results revealed that T and ND significantly increased the weight of androgen target tissues as follows: ND was more influential on seminal vesicles, levator ani-bulbocavernosus muscle (LABC) and Cowper's glands and T (at a dose of 3.0 mg/kg.bw) influenced the weight of the ventral prostate and glans penis. Serum samples analyzed for steroid hormone levels showed the presence of 17β-testosterone, 17β-estradiol and 17β-nor-testosterone, in castrated male rats injected with testosterone and nortestosterone, but no significant differences were found between thyroid responses and thyroid hormone levels. The results of this research proved the disrupting activity of T and ND when administered in high doses and the useful application of the Hershberger bioassay in the case of ND.

## Introduction

Reproductive disorders can be considered significant causes of health risk. Due to the complexity of mammalian biology, testing on animal models is currently the key to assess chemical hazards to the human reproductive system [1]–[3].

Steroid hormones are considered to be reference substances in terms of endocrine disruption, because they are mainly recognised as the most bioactive compounds in mammalian organisms [4], [5].

In males, neuro-hormonal signals transmit information to: the hypothalamic anterior-pituitary levels, the interstitial Leydig cells, Sertoli cells and to the germinative seminal epithelium. Consequently, fertility and fecundity can be inhibited by altering the function of any of these levels that concern the male genitalia and can be important in assessing the risk associated with the use of anabolic substances administered directly to humans, or used on livestock destined for human consumption, through their long-term effect [6]–[11].

The US Environmental Protection Agency (US EPA) has formed the Endocrine Disrupter Screening and Testing Advisory Committee (EDSTAC), who proposed a screening and testing strategy by focusing on androgenic, estrogenic and thyroid hormone systems [12].

Androgenic hormones are substances with a *sterol* structure, consisting of 19 carbon atoms, which are involved in the development and maintenance of primary and secondary sex characteristics in males. These hormones can cause both physiologic and anabolic action; the most important natural androgen, testosterone (T), is considered to have the lowest androgenic-anabolic activity. To maximize the effectiveness of the anabolic steroids, the structure of testosterone was subjected to various changes, like: esterification of the 17-β-hydroxyl group or alkylation in the 17th position, or carbon α position 1, 2, 9, or 11 in the steroid structure. From these compounds, nortestosterone decanoate (ND) is an anabolic androgenic steroid analogue of testosterone that belongs to the *estran* derivatives, which unlike testosterone do not possess the methyl group at C10 and the 17-β-hydroxy group. ND is esterified; hence the ability of this compound to have a prolonged action, with both favourable and undesired effects. Nortestosterone decanoate is generally synthesized chemically but can also be naturally found in some mammals. Compared to testosterone, nortestosterone-decanoate has a stronger anabolic capacity, about five times higher than T, but with a reduced androgenic activity and with more toxicological “features”. These features have been demonstrated in some animal bioassays and explained by some receptor binding studies. Although until now, information on ND overdosage management or studies about its carcinogenic or genotoxic effects on animals, are very sparse [8], [13]–[15].

Nortestosterone decanoate has many therapeutic properties; for example: in the case of postmenopausal osteoporosis [16], on weight and lean body mass in HIV-infected humans [17] and in the treatment of prostate cancer or benign prostate hyperplasia [18], but unfortunately, the endocrine disruption activity of all widely used anabolic substances including ND, through their direct action or by their cumulative effect, can also be a problem, inhibiting fertility by altering the reproductive function of the male genitalia. What is yet known is that the prolonged treatment with nandrolone in males leads to an altered androgenic function; therefore, the risk assessment is considered an important issue to establish the extent of these alterations [6], [14].

In this respect, the *in vivo* Hershberger assay was chosen to be used for the first part of this study, a test proposed by the Organization for Economic Co-operation and Development (OECD) and the Endocrine Disruptor Screening and Testing Advisory Committee (EDSTAC), to evaluate compounds that have the potential to act as androgens or anti-androgens, and has been extensively used by the pharmaceutical industry for this purpose [19]–[22]. This test is a short-term screening assay using accessory tissues of the male reproductive tract and is based on the weight changes of five main androgen-dependent tissues: ventral prostate, seminal vesicles (plus fluids and coagulating glands), levator ani-bulbocavernosus muscle (LABC), paired Cowper's glands and the glans penis in castrated prepubescent male rats. A statistically significant increase (androgenic) or decrease (ant androgenic) in the weight of at least two, out of these five tissues, will indicate a positive response in this bioassay [19].

In the recent years the rat bioassay-Hershberger has undergone a comprehensive program of validations. Until now through this bioassay, testing methods were validated for: testosterone propionate, synthetic androgens (as trenbolone acetate and methyl testosterone), a natural inhibitor of androgens (dihydrotestosterone) and a synthesis one (finasteride), other bioassays (including here for ND and other androgen compounds) being expected [1], [23]–[25].

The aim of this study was to complete the existing information about the disruptor activity of ND in an animal model based on the following: (a) weight increase of the androgen target tissues, respecting the Hershberger methodology; (b) the 17α and β-testosterone, 17 α and β-estradiol and 17 α and β-nortestosterone levels using the GC-MS/MS technique and (c) the serum free thyroxine levels (T_4_).

## Materials and Methods

The study was performed in compliance with good laboratory practice; in accordance to the European Convention principles for the protection of vertebrate animals used in experimental and other scientific purposes, adopted in 1986, in Strasbourg [26] and the 2010/63/EU Directive of the European Parliament and of the European Council adopted on 22 September 2010, [27] on the protection of animals used for scientific purposes, in accordance with Romanian law for animal experimentation and having the acceptance of the Scientific Ethic Committee of the Faculty of Veterinary Medicine Timisoara [28].

### Animals

Certified healthy animals were purchased from the authorized biobase of “Victor Babeş” University of Medicine and Pharmacy from Timisoara, RO. The animals were acclimatized for seven days and kept in standard cages at controlled temperature and humidity. For this purpose, the animals were housed in polycarbonate cages with 750×720×360 mm (L × w × h) dimensions, and wood shavings were used as bedding. The environmental temperature was maintained at 20±2°C and relative humidity of 55±10%. During acclimatization period, the light cycle was 12 h light and 12 h dark. A non-sterile pellet diet (Biovetimix, code 140–501, Romania) and water were offered *ad libitum*.

For the experiment, 30 prepubescent Wistar male rats were used with an initial body weight between 245 and 290 g, aged between 35 and 38 days. From the start to the end of the experiment, the animals were grouped (Testosterone 1 = T1; Testosterone 2 = T2; Nortestosterone 1 = ND1; Nortestosterone 2 = ND2; Control = C) and housed in five cages (six animal/cage) upon them treatment.

Due to their well studied andrologic characteristics and because this species is commonly used for reproductive toxicological studies, rats are preferable to mice and hamsters [29]. A bilateral orchidectomy, similar to the rabbit castration procedure, was performed (as presented in Figure S1 in File S1) [30].

After castration, all animals fully recovered in seven days.

### Treatment protocol

After castration and full recovery, the rats received androgen doses by i.m. way. The administration started when the rats were 49 days old. The animals were injected daily at 14.00 pm for 10 consecutive days, following within 24 h, respecting the protocol presented in Table 1.

**Table 1:** Protocol of substances administration.

Group	Rats/group	Drug/Producer	Dose/rat	Frequency/period/way
**Testosterone 1 (T1)**	**6**	Aquatest (Balkan Pharmaceuticals Moldova) testosterone aqueous solution (50 mg/ml)	1.5 mg/kgbw.	daily, for 10 days/i.m.
**Testosterone 2 (T2)**	**6**	Aquatest (Balkan Pharmaceuticals Moldova)	3.0 mg/kgbw.	daily for 10 days/i.m.
**Nortestosterone 1 (ND1)**	**6**	Deca Durabolin (Balkan Pharmaceuticals Moldova) nandrolone decanoate oily solution (200 mg/ml)	1.5 mg/kgbw.	daily for 10 days/i.m.
**Nortestosterone 2 (ND2)**	**6**	Deca Durabolin (Balkan Pharmaceuticals Moldova)	7.5 mg/kgbw.	daily for 10 days/i.m.
**Control (C)**	**6**	White sesame oil (Manicos Romania)	0.10 ml/rat	daily for 10 days/i.m.

The commercial androgens used in our experiment were testosterone aqueous suspension (Aquatest) and nortestosterone nandrolone decanoate oily solution (Deca-durabolin, C_28_H_44_O_3_ or 3-oxo-estr-4-en-17β-yl decanoate), purchased from Balkan Pharmaceuticals Ltd., Moldova. For control group administrations, white sesame oil purchased from Manicos, Romania was used.

24 h after the last administration, the rats were euthanized and examined accordingly to the OECD 441/2009 standard procedure during necropsy (OECD, 2011). The method of euthanasia used was overdosing with anaesthetic agents, using the following compound: Ketamine (300 mg/kg.bw.) + Xylazine 30 mg/kg.bw. [31].

#### Androgen target tissue sampling

The five androgen-dependent tissues: ventral prostate, seminal vesicles, levator ani-bulbocavernosus muscle (LABC), bulbourethral glands (Cowper), and glans penis were harvested (as presented in Figure S2 in File S1). The excision was performed carefully with the removal of all fat and adjacent tissues, followed by weighing of the fresh (unfixed) tissues. They were handled properly in order to avoid any drying or fluid loss, which could have led to significant errors.

### Hormone analysis methodology

#### Sample collecting

For accurate data and to avoid changes due to any other external causes, the samples for serum levels of α and β-testosterone; α and β-estradiol and α and β-nortestosterone determination, were collected per each group and after the completion of the treatments, at the same day and time interval from 7 to 8 in the morning. Blood was collected by cardiac puncture, before euthanasia, from all castrated vehicle-treated and hormone-treated rats and allowed to clot at the room temperature for 2 h. The serum was collected, centrifuged at 2000 rot/min for 10 min and the clear supernatant was used for the hormone analysis.

#### Chemicals

All chemicals and reagents were of high purity. The derivatization reagent consisted of MSTFA/ammonium iodide/dithiothreitol (1000∶2∶4, v/w/w). Standards of α-estradiol; β-estradiol; α-testosterone; β-testosterone; α-nor testosterone; β-nor testosterone and internal standard (deuterated molecules) of β-estradiol-d3; testosterone-d2; nortestosterone-d3 were obtained from the RIVM Bank of Reference Standards.

#### Extraction

5 ml of serum were extracted using a 10 ml mixture of tert-butylmethylether/petroleum ether (30∶70, v/v) in a glass tube, by shaking on the Vortex for 3 min. After the separation phase, the extracted solution was frozen at −18°C, for 1.5–2 h. Then, the ether layer was decanted into a glass tube and evaporated at 60±2°C under a nitrogen stream. The residue was dissolved in 3 ml of water, and then the sample was extracted twice with 5 ml of pentane. The pentane layer was transferred into a 15 ml glass tube and evaporated. The extract was then dissolved in 0.5 ml of ethanol and transferred into a derivatization vial. The ethanol was evaporated until dry.

#### Derivatization

The residues were redissolved in derivatization (25 µl MSTFA). After one hour of derivatization at 60±2°C, the reagent was evaporated and the residue dissolved in 25 µl isooctane.

#### GC-MS analysis

The method utilized to detect the estradiol, testosterone and serum testosterone used, was tandem GC-MS/MS (gas chromatography coupled to mass spectrometry) with a selected ion monitoring mode after electron ionisation, which was considered to be the most specific and sensitive for this group of compounds, accredited and accomplished by the National Reference Laboratory for Steroid Residues Determination, Constanţa, Romania in 2010). This method involves only a liquid-liquid extraction of the sample with tert-butyl-methyl-ether/petroleum ether (30∶70, v/v) and stage of derivatization with N-methyl-N-trimethylsilyl-trifluoroacetamide (MSTFA) [32].

The chromatographic separation was achieved using a Factor Four VF-5ms capillary column (30 m, i.d. 0.25 mm, and 0.25 µm film thickness), a constant flow of 1.5 ml helium/min. Injection pulse split less mode at 250°C was applied, injection volume 2 µl. The oven temperature was kept constant at 80°C for 1 min and was increased, 20°C per minute, up to 300°C and was kept constant at this temperature for 2 min. The EI ionisation mode was used. The calibration curve was achieved at 10.0; 7.5; 5.0; 2.5 and 1.2 ng, respectively. The internal standards were introduced to each sample at the 50 ng/L concentration. The minimum detection limits (MDL) for the analysed steroids were: α-estradiol: 0.07 ppb; β-estradiol: 0.05 ppb; 17-α-19-nortestosterone: 0.22 ppb; 17-β-19 nortestosterone: 0.26 ppb.

#### Determination of serum free thyroxin

The determination of serum free thyroxin was performed using a Free T4 8200 Architect analyzer, through Chemiluminescent Microparticles Immuno Assay (CMIA Abbot). The analysis was performed by Bioclinica laboratories, Timi?oara, RO. The principle of the CMIA method is based on a two-step immunoassay for the presence of T_4_ in the serum by chemiluminscence. In the first phase, samples were combined with paramagnetic microparticles labelled anti-T_4_. In the sample, T_4_ bound to these micro-particles. In the second phase, after washing, an acridinium conjugate labelled T_3_ was added; then, chemical reaction releasing solutions, were introduced into the sample. The resulting chemiluminescent reaction was measured in relative light units. There is an inverse relation between T_4_ and the relative light units identified by the optical system Architect [33].

### Statistical analysis

Statistical analyses were performed using the descriptive Anova test with Bonferroni correction (two-way ANOVA with Bonferroni post-test for the target organs and one-Way ANOVA with Bonferroni post-test for thyroxine) (Graph Pad Prism 5.0, San Diego USA). Differences were considered to be significant when: P<0.05, P<0.01 and P<0.001.

## Results

### (a) Testosterone and nortestosterone decanoate impact on the weight of the target tissue

#### Clinical observations

**Figure 1:**
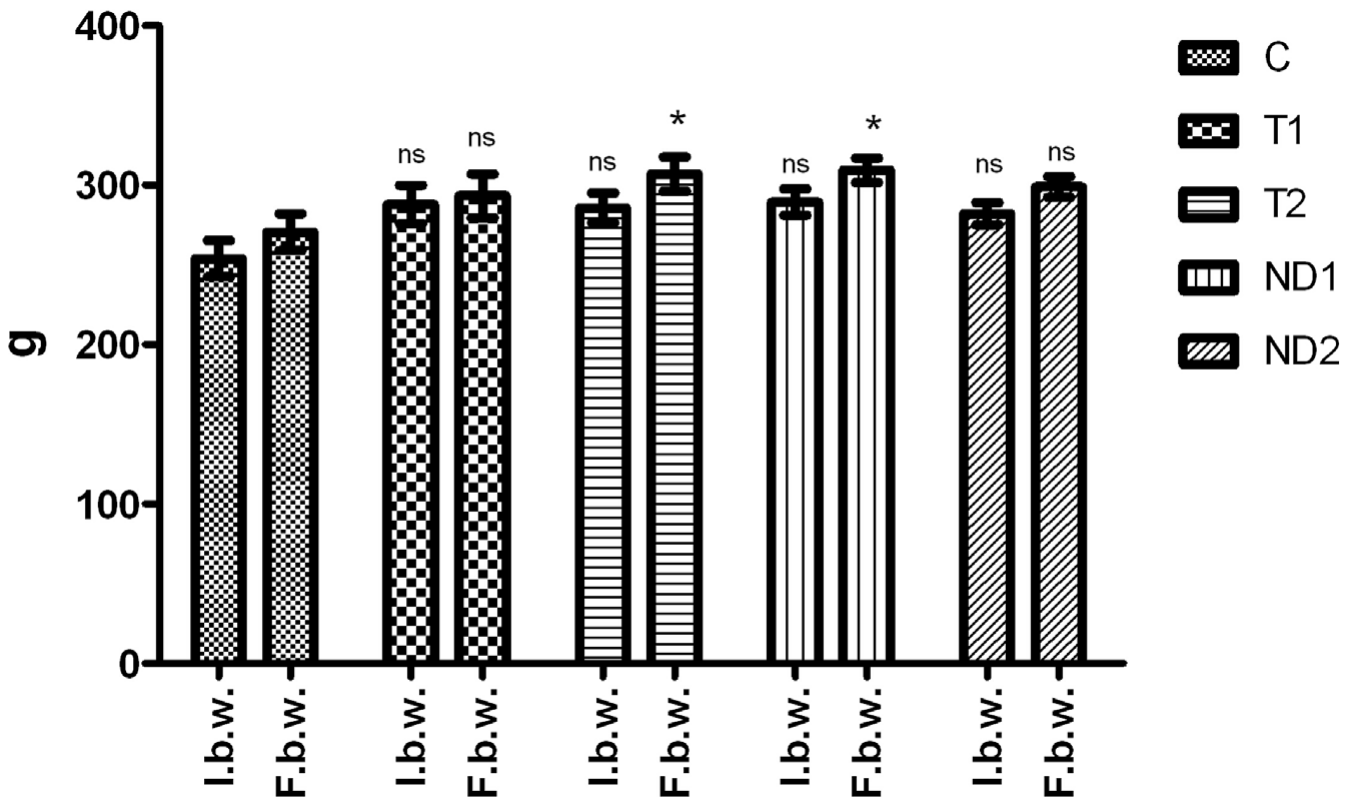
The body weight in the experimental groups (g) before and after the administration of androgen substances.

During the experiment, no fatalities occurred. The treated animals supported well the very small amounts of administrated substances, during this 10 day period, no swelling or any other general or local alterations were observed. The appetite and normal behaviour of the animals were observed in all situations. The body weight in the experimental groups before and after the administration of androgen substances is presented in figure 1.

**Figure 2:**
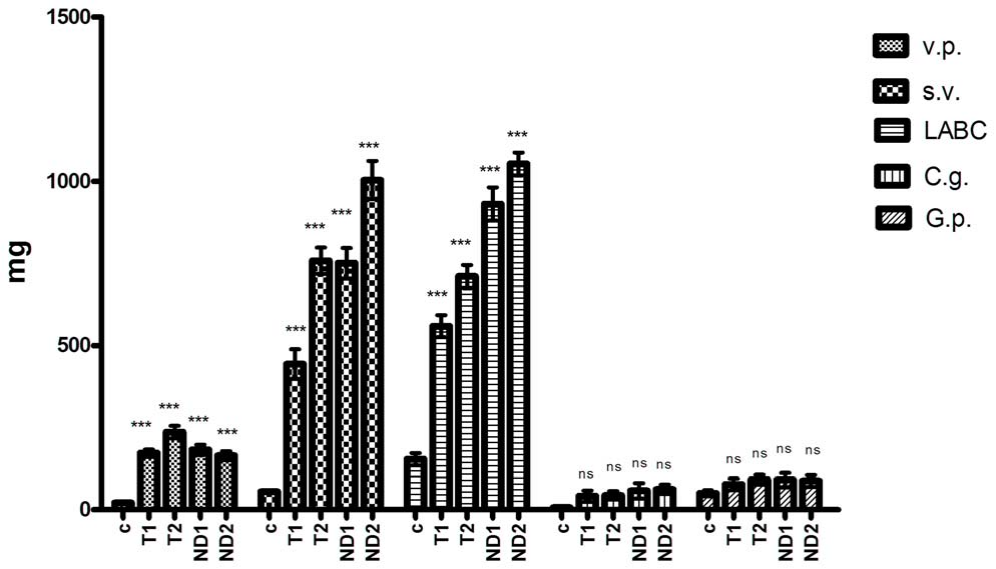
Effects of testosterone and nandrolone decanoate on the weight of accessory sex tissues.

The effects of T and ND on the weight of the accessory sex tissues were compared to the Control group. The results were summarized with the average weight of the target tissues in castrated rats, shown in figure 2 (and figures S3 to S7 in File S1 that are presenting the evolution of organs weight as presented in the SI document).

Our results after the administrations showed that the body weight of rats from the experimental and control groups varied between 270.5±28.22 g for the C group and 309.33±18.62 g for the ND1 group. The registered weight gain was between 5.97% and 7.41%.

The average weight of the *ventral prostate* (mg) decreased in this order: T2>ND1>T1>ND2>C. Extreme differences (P<0.001) between the average weight of the ventral prostate compared to the C group were recorded. No significant differences (P>0.05) were recorded between groups T1/T2, while there were no significant differences between groups T2/ND2. Comparing statistically the ventral prostate weight between groups T1/ND1, T2/ND1, T1/ND2, ND1/ND2, the registered differences were not significant (P>0.05).

Concerning the weight of the *seminal vesicles*, the descending order of the experimental groups was as follows: ND2>T2>ND1>T1>C. The seminal vesicle weight values in all experimental groups were extremely significant (P<0.001), higher than the ones measured in the C group. Comparing all groups, we found significant difference in all cases (P<0.001), apart from the groups T2/ND1, where the differences were not significant (P>0.05).

Regarding the influence of exogenous administration of ND on *LABC* weight, compared to the action of T, our results highlighted that LABC weights were significantly higher (P<0.001) in the experimental groups compared with the Control group. ND produced a greater increase in weight when compared to T at all used doses. The differences were extremely significant (P<0.001) when the groups T1/ND1, T1/ND2, T2/ND1, T2/ND2 and T1/T2 were compared and significant (P<0.01) when groups ND1/ND2 were compared.

In the case of *Cowper*'*s glands*, the ND administrations led to a higher weight gain in comparison to T, regardless of the dose used. The weight values of the Cowper's glands, in the case of T and ND groups were not significant (P>0.05).

The weight values of the *glans penis* in the experimental groups, compared to the control group or comparing between groups revealed that, the differences were not significant (P>0.05).

Our study revealed that nortestosterone decanoate produced the most significant weight gain concerning in order: the seminal vesicles, LABC and Cowper's glands in each dose (see figure 2) and testosterone in large doses affected the growth of: ventral prostate and glans penis.

### (b) Testosterone and nortestosterone decanoate impact on serum hormone levels

Testosterone and nortestosterone decanoate impact on serum hormone levels of some individual samples from the experiments are presented in figures 3–11.

**Figure 3:**
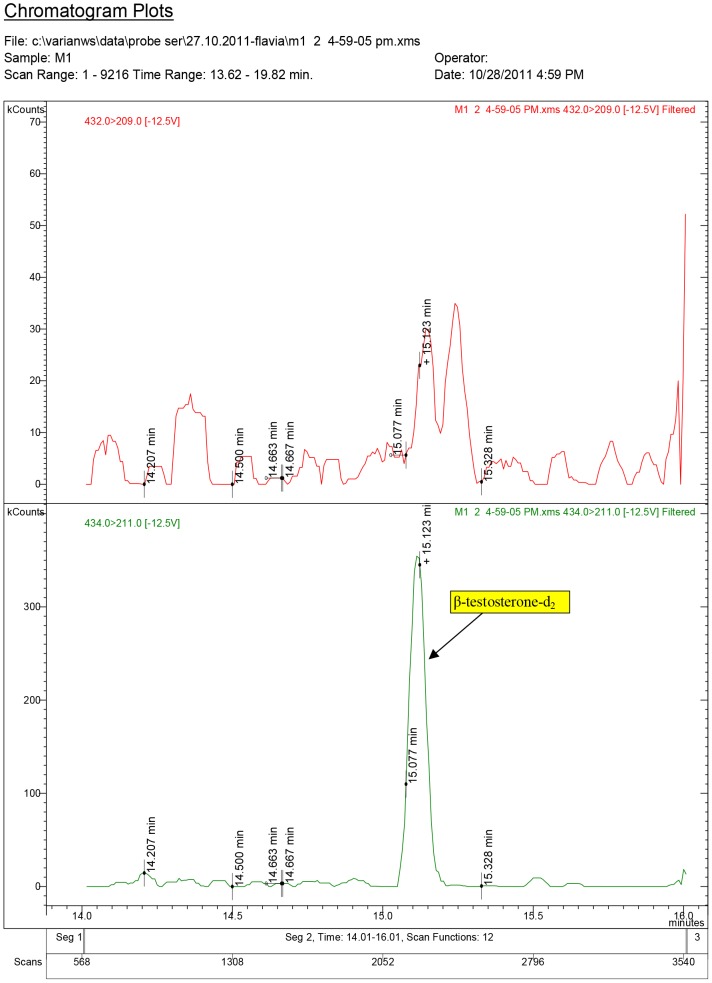
Diagnostic ions for testosterone in the Control group (injected with white sesame oil, 0.10 ml/rat).

**Figure 4:**
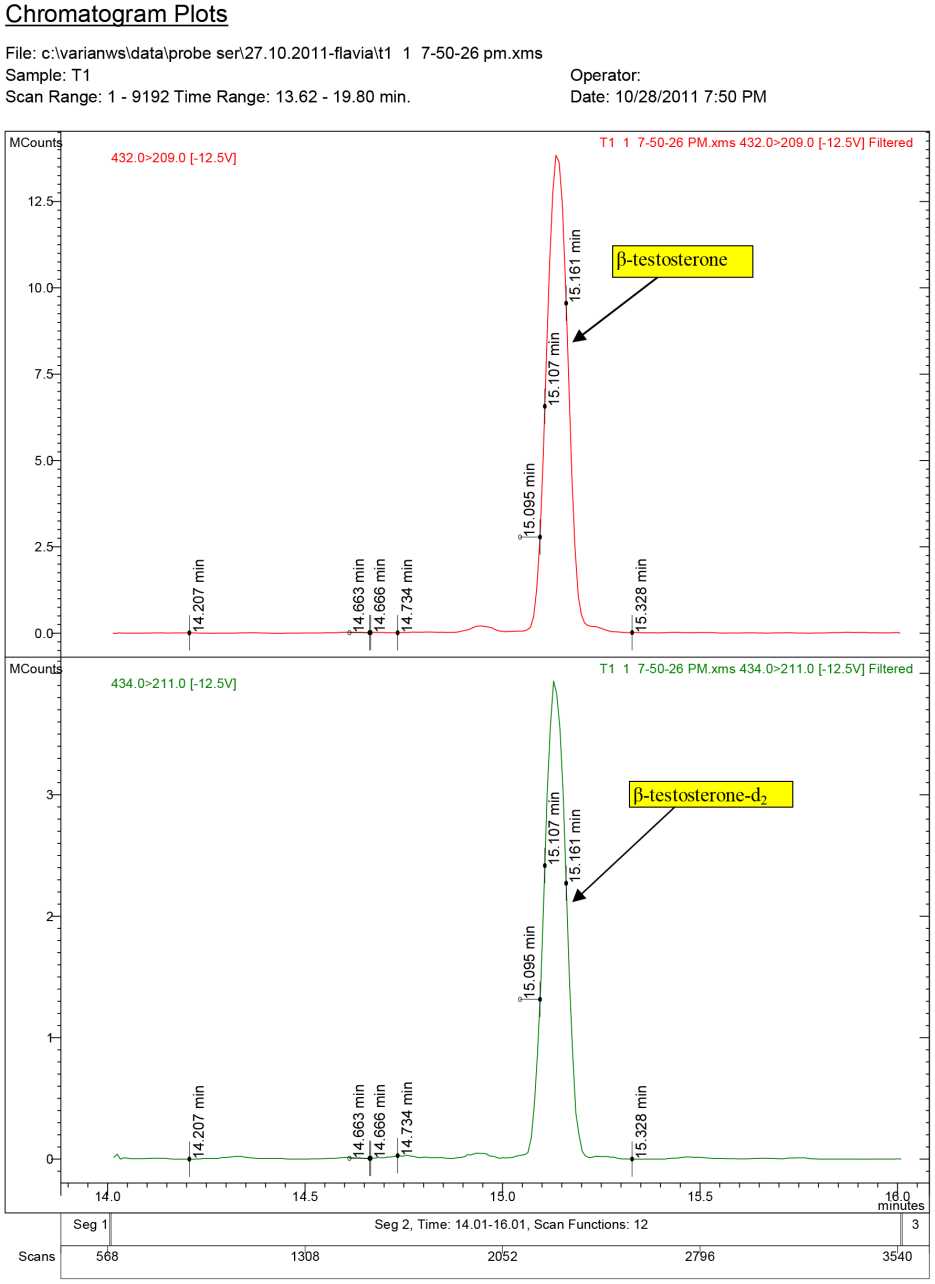
Diagnostic ions for serum sample no. T1, where 17β-testosterone was quantified at 4.40 ng/ml (ppb) (group T1, dose: 1.5 mg/kg.bw).

**Figure 5:**
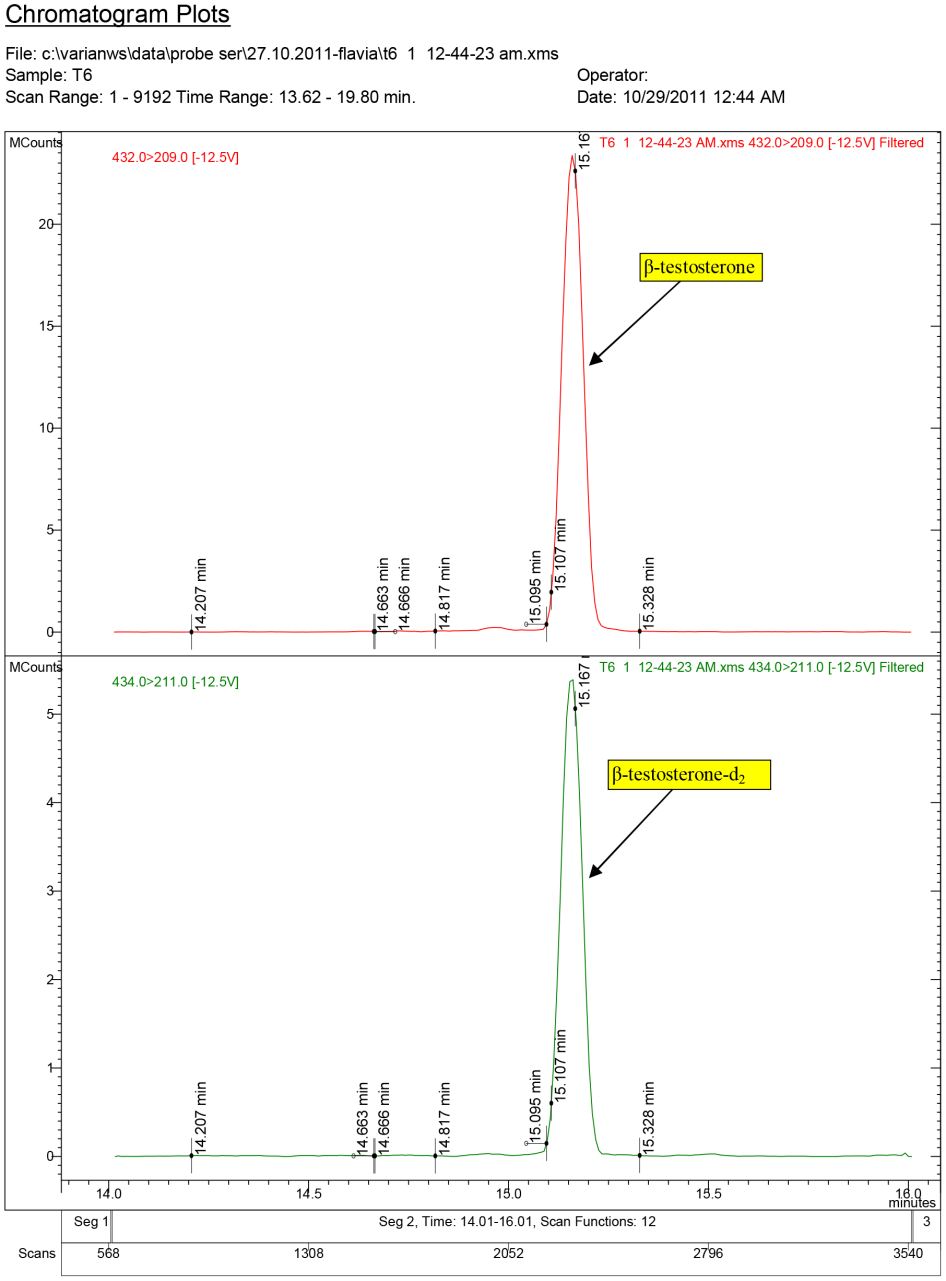
Diagnostic ions for serum sample T6, where 17β-testosterone was quantified at 7.04 ng/ml (ppb) (group T2, dose: 3.0 mg/kg.bw).

**Figure 6:**
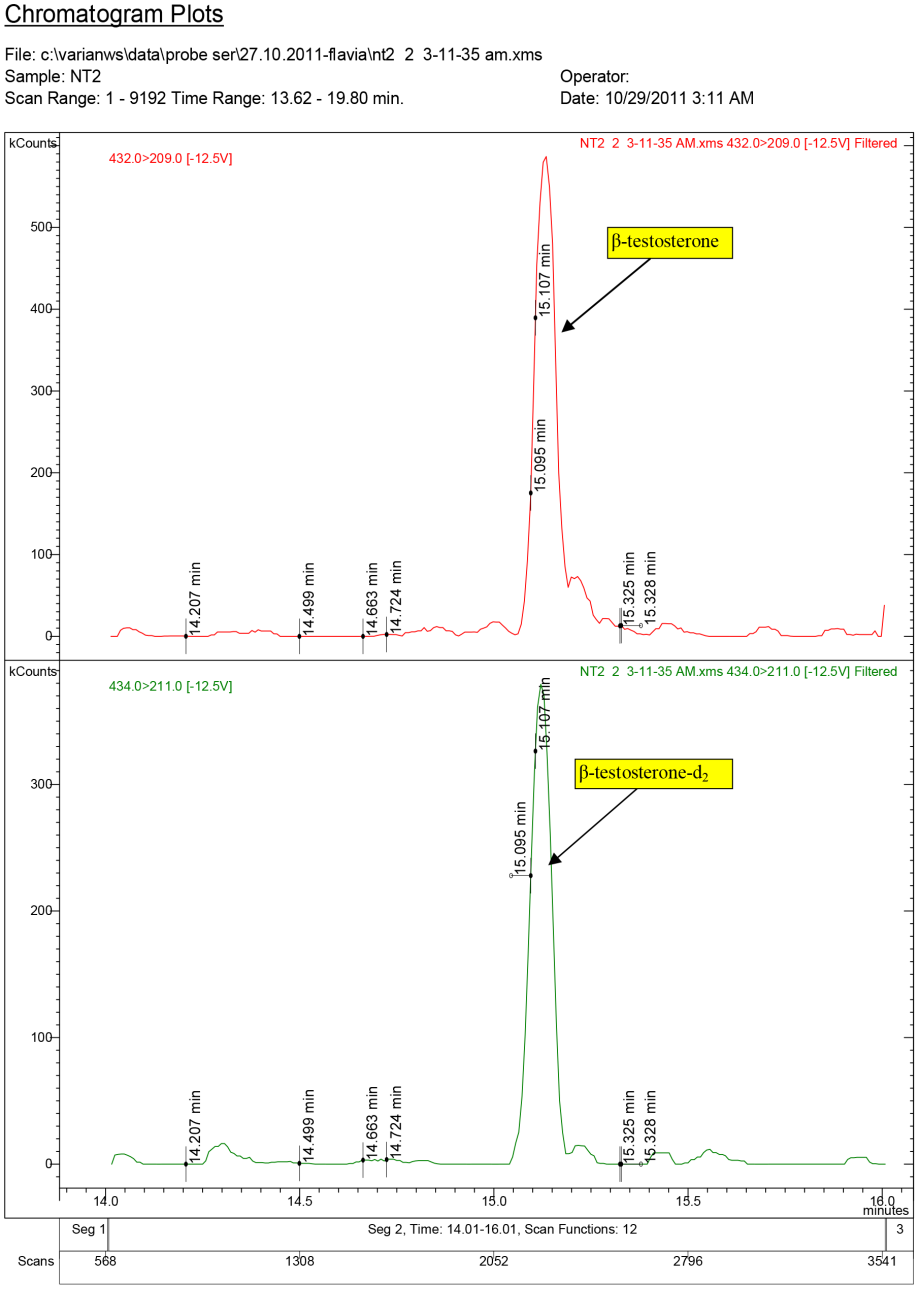
Diagnostic ions for serum sample NT2, where 17β-testosterone was quantified at 2.29 ng/ml (group ND1, dose: 1.5 mg/kg.bw).

**Figure 7:**
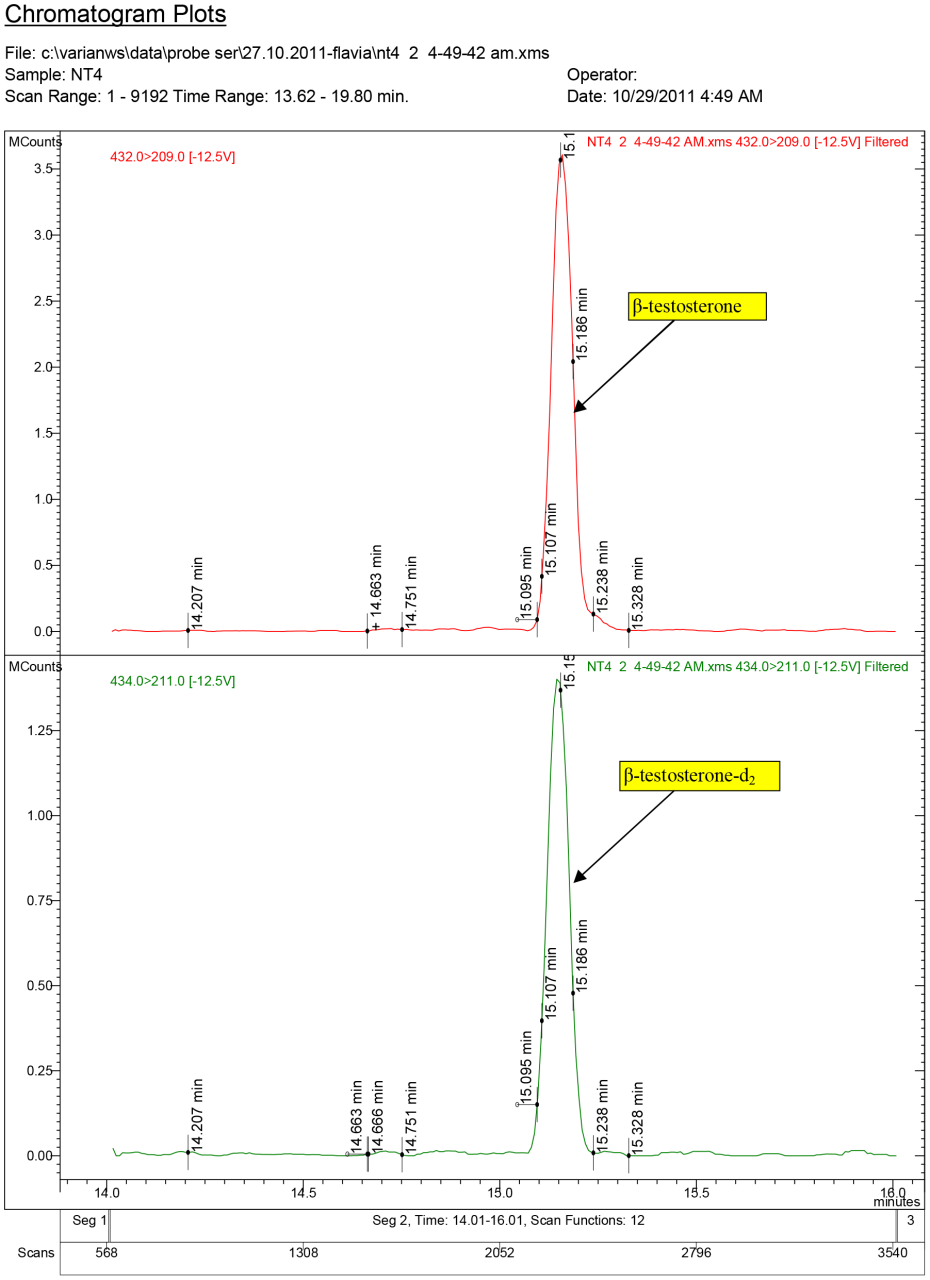
Diagnostic ions for serum sample NT4, where 17β-testosterone was quantified at 4.83 ng/ml (group ND2, dose: 7.5 mg/kg.bw).

**Figure 8:**
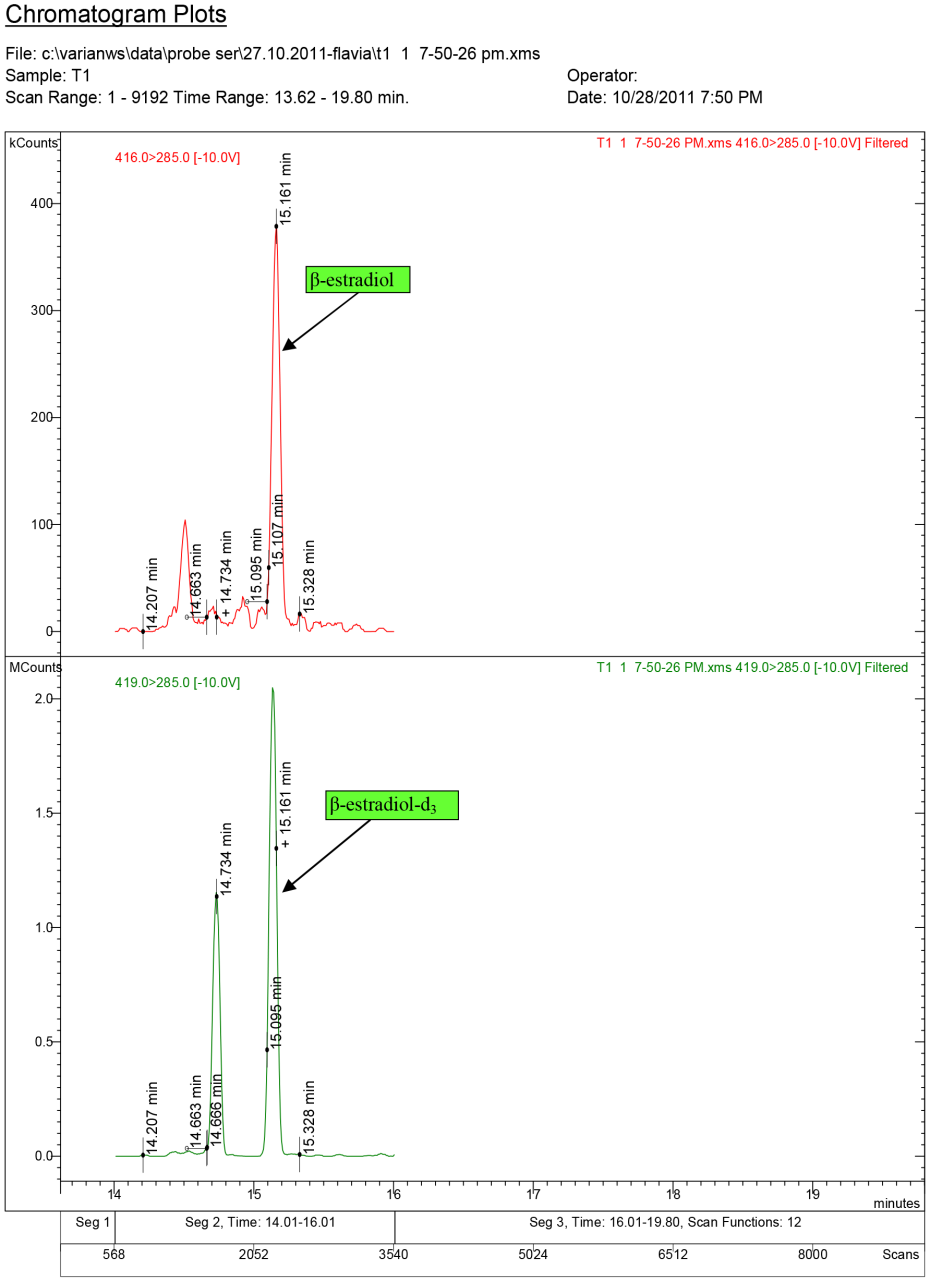
Diagnostic ions for serum sample T1, where 17β-estradiol was quantified at 0.11 ng/ml (group T1., dose: 1.5 mg/kg.bw).

**Figure 9:**
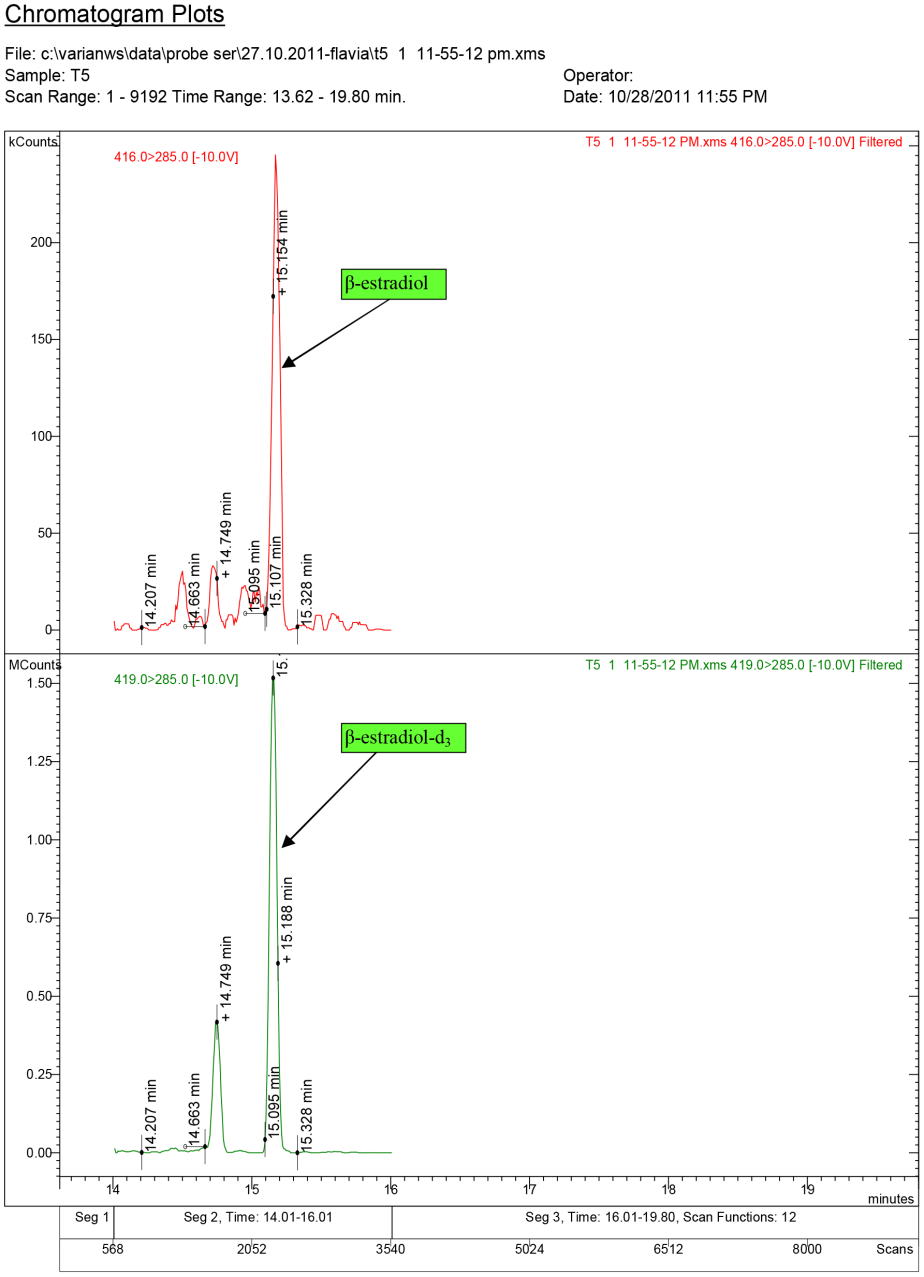
Diagnostic ions for serum sample T5, where 17β-estradiol was quantified at 0.17 ng/ml (group T2 dose: 3.0 mg/kg.bw).

**Figure 10:**
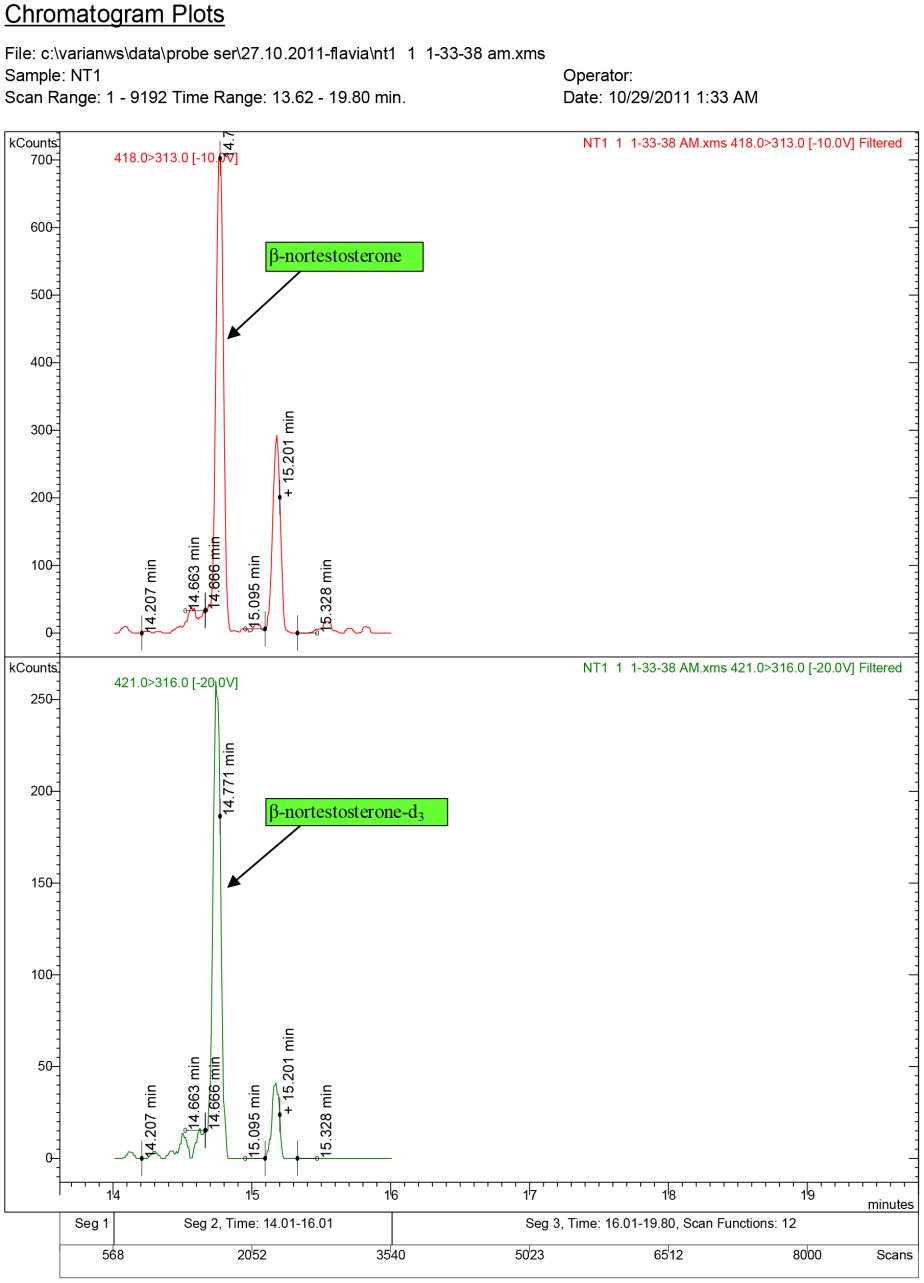
Diagnostic ions for serum sample NT1, where 17β-nortestosterone was quantified at 1.57 ng/ml (ND1 dose: 1.5 mg/kg.bw).

**Figure 11:**
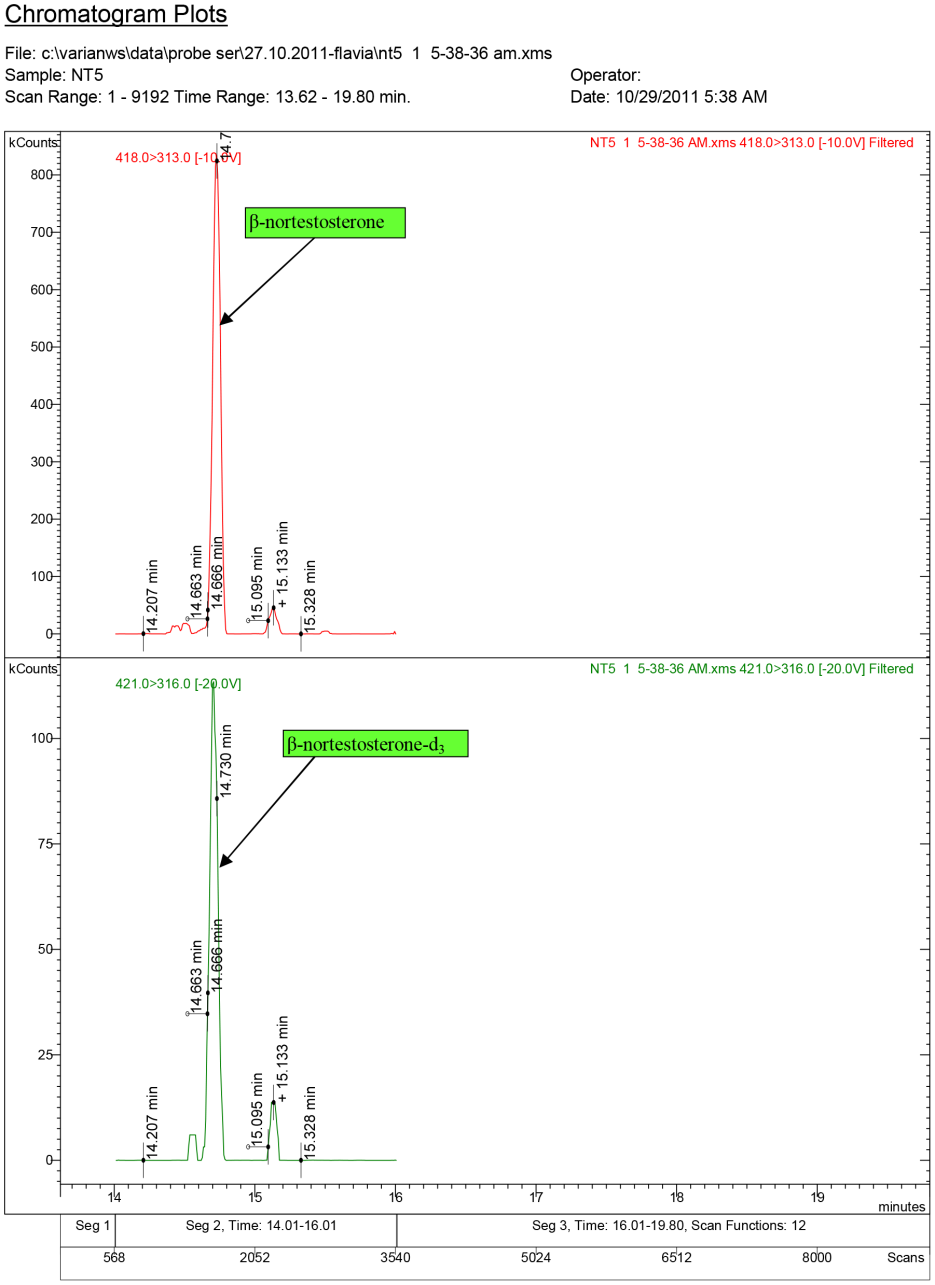
Diagnostic ions for serum sample NT5, where 17β-nortestosterone was quantified at 4.22 ng/ml (ND2 dose: 7.5 mg/kg.bw).

The chromatogram plots are presented for the standards of 17α-testosterone and 17β-testosterone. Following the interpretation of the results obtained from the serum samples, 17β-estradiol was identified only in the experimental groups receiving testosterone, as a result of its aromatization [34].

In the Control group treated with white sesame oil, testosterone content was below the Minimal Detection Limit (MDL).

In table 2, the mean serum concentration values for the hormones found in the groups studied (ng/ml) (ppb) are presented.

**Table 2:** Mean serum concentration values of hormones found in the studied groups (ng/ml  =  ppb).

Substance	Testosterone				Oestradyol		Nortestosterone decanoate	
Statistic Parameter/group	T1	T2	ND1	ND2	T1	T2	ND1	ND2
**X±SX**	5.87±0.53	6.85±0.27	2.93±0.95	3.67±0.94	0.06±0.01	0.10±0.02	1.78±0.17	3.80±0.10
**SD**	1.30	0.67	2.33	2.30	0.03	0.04	0.41	0.26
**confidence level 95%**	0.94	0.94	1.53	1.53	0.03	0.03	0.31	0.31

Each value is mean ±standard deviation of six animals.

The obtained results highlighted that in the T1 group, which received testosterone aqueous solution at a dose of 1.5 mg/kg.bw, the mean value of 17β-testosterone was found in a concentration of 5.87 ng/ml.

In the T2 group, which received T aqueous solution at a concentration of 3.0 mg/kg.bw 17β-testosterone was identified and quantified at a mean concentration of 6.85 ng/ml.

For the groups treated with ND, quantified molecules were: 17β-nortestosterone and 17β-testosterone. Of the two isomers of nortestosterone, α and β-nortestosterone, only β-nortestosterone was identified and quantified in serum samples from ND1 and ND2 experimental groups.

In ND1 group, which received a ND doze of 1.5 mg/kg.bw, 17β-testosterone was measured having a mean value of 2.93 ng/ml, while in the ND2 group (7.5 mg/kg.bw), 17β-testosterone was recorded at a concentration of 3.67 ng/ml. The mean value of 17β-nortestosterone for ND1 group was of 1.78 ng/ml and of 3.80 ng/ml for ND2 group.

Our study highlights that in serum samples from the experimental groups, 17β-testosterone concentrations showed no significant difference (P>0.05) between T1 and T2 groups and between ND1 and ND2 groups, but comparing the T1 group with the ND1 and the T2 with the ND2, there were significant differences (P<0.05), testosterone being found to have higher values.

The mean of 17β-estradiol concentration was quantified as 0.06 ng/ml for T1, and 0.10 ng/ml for the T2 group. Estradiol concentrations showed no significant differences (P>0.05) between the two experimental groups treated with T, but the 17β-nortestosterone concentration in serum samples collected from the ND2 experimental group were highly significant (P<0.0001), higher than the concentration of 17β-nortestosterone from the ND1 group samples. When comparing the groups ND1/ND2, the differences were extremely significant (P<0.0001).

### (c) Testosterone and nortestosterone decanoate impact on levels of serum free T4

**Figure 12:**
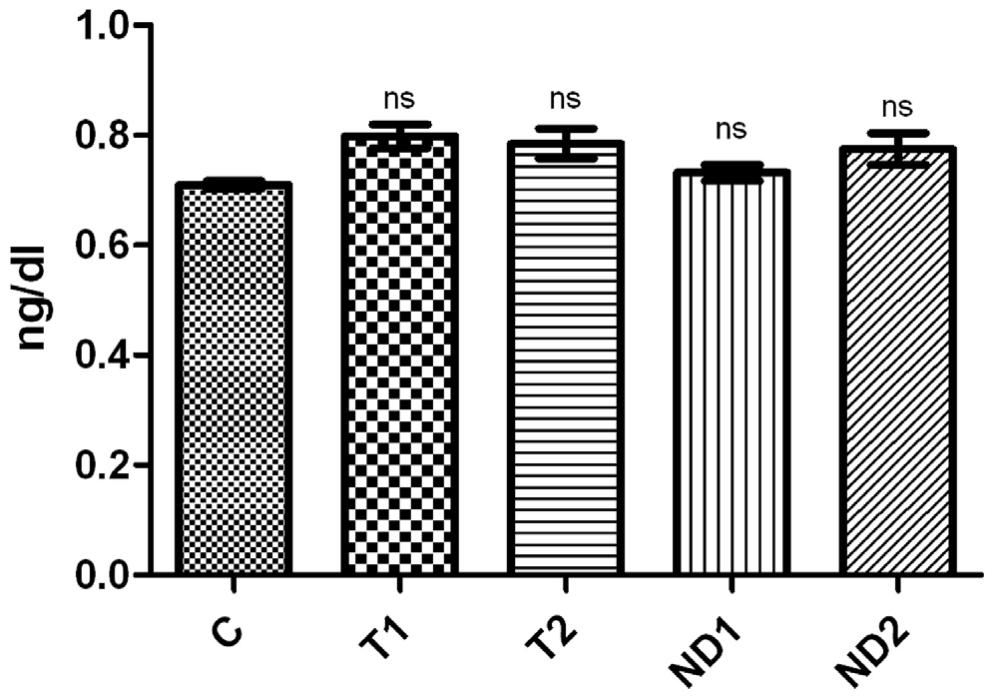
Effect of T and ND on serum free T_4_.

The T_4_ values obtained from castrated males in the C group and in the groups treated with T and ND are shown in figure 12.

Our results registered a media concentration of 0.71 ng/dl in the C group, 0.80 ng/dl in T1 group, 0.78 ng/dl in T2 group, 0.73 ng/dl in ND1 group and respectively 0.78 ng/dl in ND2 group. Comparing the groups, no significant differences were found.

## Discussion

The significant role played by endogenous and exogenous steroids in male gametogenesis has opened a vast research area into andrology. In reproductive target tissues, testosterone can be considered to be a pro-hormone, readily converted by 5α-reductase to the more potent androgens (dihydrotestosterone). In addition, some synthetic substances (e.g., 5α-reductase inhibitors) inhibit the conversion of testosterone to dihydrotestosterone, the most potent natural steroid in androgen target tissues. Such substances have the potential to produce adverse health effects, regarding the reproductive function or body development [35], [36].

Most of the studies on the subject, have demonstrated the effect of ND on the testis, and altering the reproductive function of male genitalia can inhibit fertility and fecundity; ND can easily become an endocrine disruptor [37]. We agree with other author who demonstrated that prolonged treatments of ND in males may lead to altered testicular morphology [38], a permanent decrease of testosterone secretion [6] or reduction of sperm quality [11].

We consider that the study of ND, another synthetic anabolic androgenic steroid analogue of T, was justified because of its role as an endocrine disruptor, which can be of considerable importance in medicine. In some cases, these compounds are associated with direct carcinogen toxicity. In the present study, the administration of T and ND at two different doses to young animals resulted in a pattern of effects on the target organs that could be used to highlight their interaction with the steroidal metabolism.

Considering the influence of T and ND from exogenic sources on the body weight of rats, our results highlight that except in the T1 group, where the weight gain was minimal (1.0%), the other experimental groups that had no significant differences compared to the control group, were not recorded. Similar results were reported by Hale *et al.*, 1972, they showed that the administration of testosterone propionate to castrated male rats at doses ranging from 0.05 to 0.80 mg/animal/day compared to nandrolone phenyl propionate, influenced the final body weight to a very small extent. [39]. Another study, Freyberger *et al.*, 2005, argued the increase in body weight of the animals treated with testosterone propionate, and the weight reduction in those treated with trenbolone [24].

Our results concerning the reproductive alterations through the increased weight of the target tissues observed in animals treated with T and ND concur with the aforementioned studies.

Some authors suggest that the deleterious effects of exogenous ND on the reproductive tract would be due to a disruption of the feedback regulation on the way of the hypothalamic-pituitary-gonadal axis, after administration [37], [38].

Kumar *et al.*, 2008, investigated the function of endocrine disruptors, their influence on reproductive processes and systemic toxicity in castrated and uncastrated male rats using a 20 days Hershberger protocol. The results revealed that for uncastrated males from the control group, the mean testosterone obtained was 7.5 ng/ml, while for castrated males from the treated group, this value was much greater (10.40 ng/ml) this dynamics being observed also by our collective after a 10 days protocol in castrated males [40].

Kennel *et al.*, 2004, reported similar results regarding the content of 17β-testosterone in the plasma, when testosterone propionate and methyl-testosterone were used [1]. A previous study conducted to measure serum concentrations of ND and T in a clinical trial for male fertility control following intramuscular injection of ND serum, demonstrated that the concentration of 17-β-19-nortestosterone and 17β-testosterone, increased rapidly and reached maximal concentrations and the half-life of ND was 8 days [41]. On the other hand, Purkayastha *et al.*, 2012, observed no initial change in T levels after 10 days of ND administration, but the decrease in serum levels was found to be highly significant (P<0.01) from day 30 [42].

Following the interpretation of the results of the serum samples, 17β-estradiol was found only in the experimental groups receiving T, indicating the biotransformation of T; however, the estrogenic effect of ND was considerably lower than that of T. The content of steroid hormones in the C group was below the MDL. This may be explained as a result of castration in the prepubertal period. The experimental protocol was also aimed to demonstrate whether T and ND influence the thyroid gland in any way. T_4_ in the blood is one of the two main thyroid hormones along with tri-iodothyronine (T_3_). Thyroid hormone production is based on a feedback system of the body. When the level of T_4_ in circulation decreases, the hypothalamus releases thyrotrophic releasing hormone, which stimulates the release of TSH by the pituitary gland, TSH stimulates the thyroid to produce a greater amount of T_4_. With the increasing concentration of T_4_, the release of TSH is inhibited. In the blood, T_4_ is either free, or in bound form, the free T_4_ concentration being only 0.1% of the total hormone found in a relatively inactive status, but converted to an active hormone, T_3_, in the liver and other tissues. In rodents, the binding globulin of thyroxin is absent; the assessment of T_3_, T_4_ and TSH are inter alia, the routine tests for the evaluation of thyroid hormone homeostasis. The effects of the administration of androgenic substances on the serum and plasma constituent will increase the levels of calcium, sodium, and chlorine, and decrease those of serum thyroxine [42].

The registered rat serum free T_4_ levels from our study were between 0.25 and 5.00 ng/dl, these results being considered to be in the normal range, and are not supporting the case of the potential effect of exogenous androgens on the thyroid, after short term or unique exogenic administrations.

Our findings are in accordance with a research of Yamada *et al.*, 2004, they observed that there are no significant differences between thyroid responses by observing peripheral thyroid hormone levels, for castrated rats and castrated rat groups treated with testosterone propionate, because at least 2-6 weeks of dosing are required to observe an obvious thyroid response [21].

Based on the obtained data, we suggest that the Hershberger assay can represent an important tool for the assessment of nortestosterone decanoate synthetic steroid hormone as potential endocrine disrupter. The effects of testosterone and nortestosterone decanoate on the weight of accessory sex tissues were statistically significant compared to the control group. Concerning the effect of exogenous administration of T and ND, our results highlighted that the 17β-testosterone and 17-β-19-nortestosterone concentrations in plasma can increase with the dose of hormones.

## Conclusions

Testosterone administered in a dose of 3.0 mg/kg.bw influenced the ventral prostate and glans penis, which caused higher values than the rest of the experimental groups. In the groups treated with testosterone, the 17β-estradiol values obtained indicated biotransformation, while estrogenic values for nortestosterone decanoate were considerably lower.

Nortestosterone decanoate administered in a dose of 7.5 mg/kg.bw produced a significant weight gain of the seminal vesicles, LABC and Cowper's glands. The results demonstrate that the increase of ND dose will lead to 17β-testosterone and 17β-nortestosterone accumulation in the serum.

Concerning the direct impact of T and ND on serum free T_4_, the obtained results can be considered normal in all cases, although there are no supporting theories on the potential effect of exogenous androgens on the thyroid.

## Supporting Information

File S1 **Supporting Figures.** Figure S1, Aspects of rat's castration phases: anesthesia; fixing testicles; testicular cord ligature; healing powder application; appearance of castration wound. Figure S2, Depicts rat's androgen-dependent fresh wet tissues which increased more significantly them weight after treatment with ND: seminal vesicles (*left*) and bladder (*right*) (*right side image*); Levator ani-bulbocavernosus muscle (*middle*); Cowper's glands (*left side image*). Figure S3, Mean weight of ventral prostate (mg). Figure S4, Mean weight of seminal vesicles (mg). Figure S5, Mean weight of levator ani-bulbocavernosus muscle (mg). Figure S6, Mean weight of Cowpeŕs glands (mg). Figure S7, Mean weight of glans penis (mg).
(DOCX)
