# Supplementary material for: The Impact of Exogenic Testosterone and Nortestosterone-Decanoate Toxicological Evaluation Using a Rat Model
Source: PLoS One. 2014 Oct 10;9(10):e109219. doi: 10.1371/journal.pone.0109219 (PMC4193791; doi:10.1371/journal.pone.0109219)
Supplement: File S1 — Supporting Figures. Figure S1, Aspects of rat's castration phases: anesthesia; fixing testicles; testicular cord ligature; healing powder application; appearance of castration wound. Figure S2, Depicts rat's androgen-dependent fresh wet tissues which increased more significantly them weight after treatment with ND: seminal vesicles (left) and bladder (right) (right side image); Levator ani-bulbocavernosus muscle (middle); Cowper's glands (left side image). Figure S3, Mean weight of ventral prostate (mg). Figure S4, Mean weight of seminal vesicles (mg). Figure S5, Mean weight of levator ani-bulbocavernosus muscle (mg). Figure S6, Mean weight of Cowpeŕs glands (mg). Figure S7, Mean weight of glans penis (mg). (DOCX) [file pone.0109219.s001.docx]

**Figure S1**

| **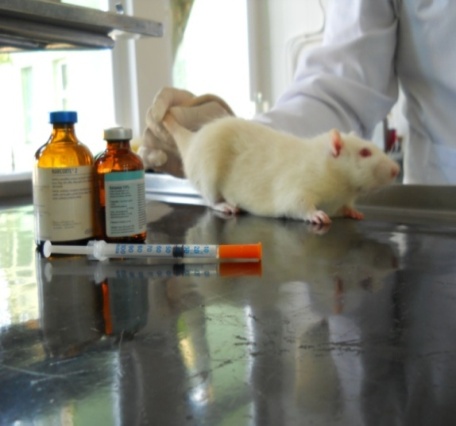** | 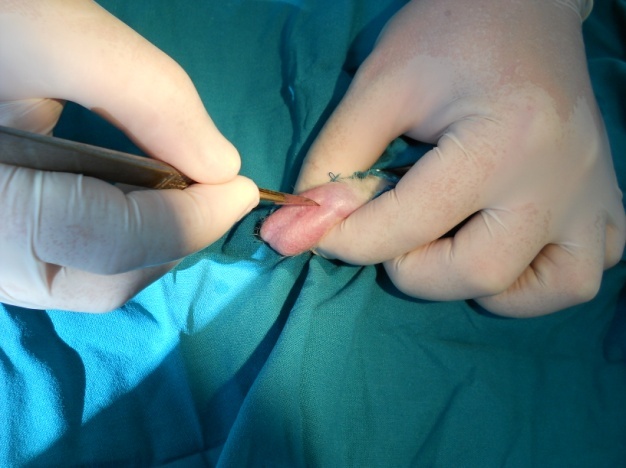 | 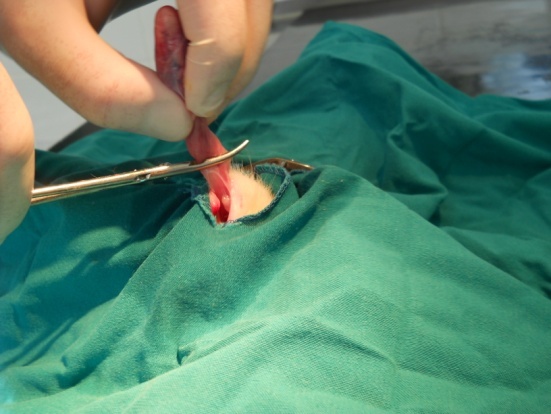 |
| --- | --- | --- |
| 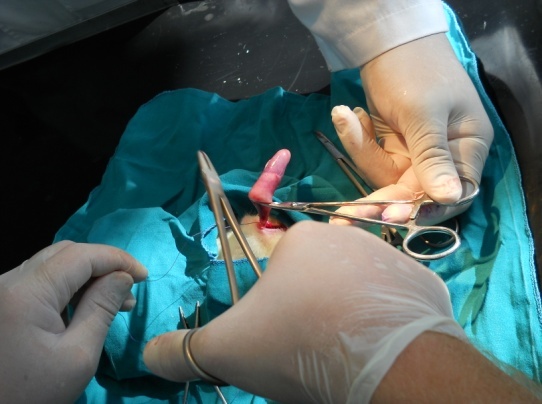 | 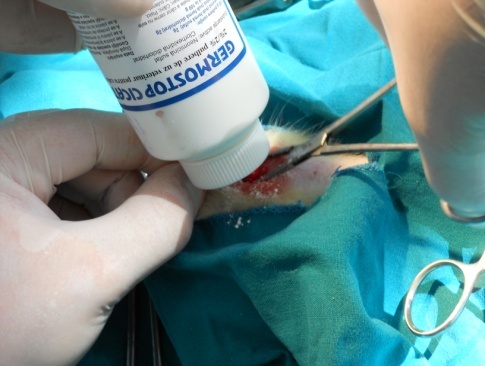 | 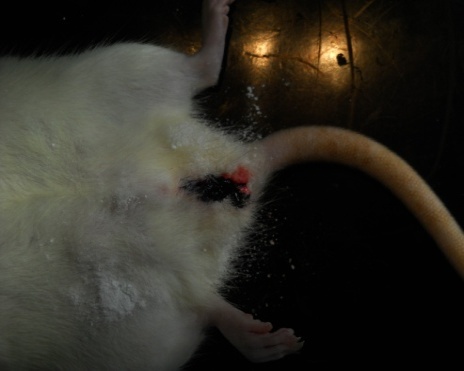 |

**Figure S1.** Aspects of rat’s castration phases: anesthesia; fixing testicles; testicular cord ligature; healing powder application; appearance of castration wound

**Legend:**

General anesthesia was performed with the association: ketamine (ketamine 10%, CP Pharma), 75-100 mg / kg.bw, and xylazine (Narcoxyl, Intervet International), 5-10 mg / kg.bw, administered by i.m. way, in the same syringe.

*Surgical technique included:*

- setting as closely of testis to the scrotal - dartoic bag;
- scrotal incision of dartoic tissue and Cowper's cellulose;
- testicle and vaginal sheath externalization in the operator plan;
- testicular cord fixing and isolation, through cellulose conjunctive tissue dilacerations;
- transfixed ligature with synthetic absorbable wire and cutting of testicular cord, distal to ligature; or ablation and haemostasys by limited torsion for both testes.
- healing powders application in castration wound.

**Figure S2**


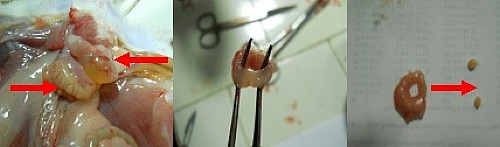


**Figure S2.** Depicts rat’s androgen-dependent fresh wet tissues which increased more significantly them weight after treatment with ND: seminal vesicles (*left*) and bladder (*right*) (*right side image*); Levator ani-bulbocavernosus muscle (*middle*); Cowper’s glands (*left side image*).

Figures S3, S4, S5, S6, S7 and S8 are presenting the evolution of organs weight:

**Figure S3. Mean weight of ventral prostate (mg)**

**Figure S4. Mean weight of seminal vesicles (mg)**

**Figure S5.** Mean weight of levator ani-bulbocavernosus muscle (mg)

**Figure S6.** Mean weight of Cowper´s glands (mg)

**Figure S7.** Mean weight of glans penis (mg)
